# Towards a unified Latin American Natural Products Database: LANaPD

**DOI:** 10.2144/fsoa-2020-0068

**Published:** 2020-06-19

**Authors:** José L Medina-Franco

**Affiliations:** 1 DIFACQUIM Research Group, Department of Pharmacy, School of Chemistry, Universidad Nacional Autónoma de México, Mexico City, Mexico

**Keywords:** chemical space, chemoinformatics, database, drug discovery, LANaPD, molecular diversity, natural sources

## Abstract

Around the world, the number of compound databases of natural products in the public domain is rising. This is in line with the increasing synergistic combination of natural product research and chemoinformatics. Toward this global endeavor, countries in Latin America are assembling, curating, and analyzing the contents and diversity of natural products available in their geographical regions. In this manuscript we collect and analyze the efforts that countries in Latin America have made so far to build natural product databases. We further encourage the scientific community in particular in Latin America, to continue their efforts to building quality natural product databases and, whenever possible, to make them publicly accessible. It is proposed that all compound collections could be assembled into a unified resource called LANaPD: Latin American Natural Products Database. Opportunities and challenges to build, distribute and maintain LANaPD are also discussed.

## Lay abstract

Due to the importance of natural products in drug discovery and other areas of science, research groups are building and maintaining compound databases of natural products. This report analyzes the compounds databases developed and published, thus far by Latin American countries and proposes the creation of a unified resource named LANaPD: Latin American Natural Products Database. The generation, curation, analysis, distribution and maintenance of LANaPD will require joint efforts from different countries. Such a goal is not straightforward and simple but it is possible and will be useful. In this report the benefits and challenges of creating LANaPD are discussed.

For centuries, natural products (NPs) have been the basis for the prevention and treatment of diseases. Up to date, NPs continue to have a profound impact in drug discovery [1]. They are compounds from natural sources or are NP derivatives that are now drug approved for clinical use. Amid the current pandemic of COVID-19, a notable example of a promising NP is chloroquine phosphate that is an analogue of the alkaloid quinine, originally extracted from the bark of cinchona trees. In addition, NPs have largely contributed to compounds, that to start compounds that are later optimized in terms of potency or pharma kinetic or pharmacodynamics properties or serve as source of inspiration to synthesize organic compounds. It is well known that the heath-related benefits are somehow associated with their structural uniqueness, diversity and complexity, as compared with other drugs from different sources [2].

Chemical informatics (also termed in the literature ‘chem[i]oinformatics’) is increasingly contributing to drug discovery at different levels [3]. For instance, a key contribution of this research discipline is designing, building and curating compound databases. Indeed, compound databases play a significant role in drug discovery and different collections, in particular in the public domain, have been reviewed. Additional major contributions of chemoinformatics to drug discovery projects are assisting screening compounds (e.g., filtering *in silico* compounds libraries to select compounds for experimental testing) and analyzing the outcome of experimental screening assays, either, in small, medium and/or high-throughput format [3]. Based on the experimental results, cheminformatics help to generate and/or refine hypothesis of the mechanism of action of bioactive molecules at the molecular level and/or build models to predict the outcome of untested compounds for example, part of a new cycle of *in silico* screening. All these and several other chemoinformatics tools have been successfully applied for organic compounds, including NPs and food chemicals [4].

Contributions of informatics to advance NP research in general, and NP-based drug discovery in particular, are increasing [5]. One of such key contributions has been the organization and analysis of chemical information of NPs, with or without biological activity, in compound databases. Over the past 5 years, several reviews of NP databases have been published [6–9]. Some of these reviews include chemoinformatic analysis of the contents, diversity and coverage of the compounds in chemical space. Of note, there has been a rapid increase in the number of publicly accessible NP databases. One of the first reviews was published in 2012 [7] that included five NPs datasets (commercial and noncommercial with chemical structures available on the web). Recently, it was released the COlleCtion of Open NatUral producTs (COCONUT) database that collects over 120 databases collecting more than 400,000 nonredundant NPs and are freely accessible [10]. As part of the global efforts, different countries around the world are analyzing the information of NPs in their countries of origin. As part of such global efforts, different Latin American countries are building their own compound databases using chemoinformatics resources.

The primary goal of this report is to discuss the recent progress of countries in Latin America to put together, curate, and analyze compound databases of NP molecules contained in their geographical region. Indeed, Latin American countries are traditionally rich in their unique biodiversity and herbal medicine has a strong tradition and use in the region. Herein, we also propose to join efforts and assemble a unified Latin American Natural Products Database (LANaPD).

## NP databases in Latin America

Thus far, Brazil, Mexico, and Panama have published NP databases. In some cases, the chemical structures are already available in the public domain and/or comprehensive analyses of their content and diversity have been released. In this section, we discuss the progress on the development of such compounds databases. For each one we describe briefly the research group and institution developing the database, the contents and number of compounds currently available, accessibility and capabilities to browse the contents and where available. We also summarize recent analysis of the chemical diversity and coverage of the chemical space and other uses.

### NuBBE_DB_

#### Developers

This public database was launched in 2013 as a joint effort of the Brazilian research groups Nuclei of Bioassays, Biosynthesis and Ecophysiology of Natural Products (NuBBE) of the São Paulo State University and the Laboratory of Computational and Medicinal Chemistry of the University of São Paulo.

#### Contents

The first release of NuBBE_DB_ contained approximately 640 compounds collected from publications of the NuBBE research group [11]. Four years later, the same group published an update expanding the number of compounds to more than 2000, thus, increasing representation of the large biodiversity in Brazil. The update also had significant enhancements to the website interface [12]. Compounds in NuBBE_DB_ are secondary metabolites of plants, fungi, insects, marine organisms, and bacteria.

Compounds in NuBBE_DB_ are annotated with chemical, biological, pharmacological and spectroscopic data. Chemical information includes international union of pure and applied chemistry (IUPAC) name, chemical structure, drug-like physicochemical properties and metabolic class. The biological information comprises species, geographical location and biological activities. The spectroscopic data includes molar mass, and nuclear magnetic resonance data.

#### Accessibility & searching capabilities

NuBBE_DB_ is accessible and searchable at the website interface. It is also available at ChemSpider and ZINC 15 where it can be found, for instance, as a NP catalog. NuBBE_DB_ has been recently included in the COCONUT database [10].

The user can download the entire database or perform online searches. It has inbuilt a broad range of searching and filtering criterions. For instance, it is possible to search by species, geographical region in Brazil, source, biological properties, chemical structure, chemical drug-like descriptors, spectroscopic data (specifically, nuclear magnetic resonance information) and bibliographic information.

#### Diversity analysis & other applications

The most recent published version of NuBBE_DB_ was analyzed based on structural diversity and complexity of the chemical structures. To this end, several chemoinformatic tools were employed. As part of the study, the contents and diversity profile NuBBE_DB_ were compared with other commercial and noncommercial NP collections whose chemical structures are freely available. The reference collections included the Universal Natural Product Database, with more than 200,000 molecules [13] and ChEMBL. It was concluded that compounds in NuBBE_DB_ are diverse in terms of molecular fingerprints, chemical scaffolds and drug-like properties. Using established chemoinformatic tools, the study supported that several compounds in NuBBE_DB_ are promising candidates for drug discovery and medicinal chemistry [14]. Interestingly, the study also revealed that 12% of the chemical scaffolds in NuBBE_DB_ are not present in ChEMBL. Also, an *in silico* ADMET profiling of NuBBE_DB_ has been published recently [15]. As discussed hereunder, chemoinformatic comparisons of NuBBE_DB_ and other NP databases in Latin America have been performed. NuBBE_DB_ has been successfully used in several drug discovery and dereplication studies as reviewed in [12].

### CIFPMA

#### Developers

Over the past few years, the Center for Pharmacognostic Research on Panamanian Flora, College of Pharmacy of the University of Panama (CIFLORPAN, for its acronym in Spanish) has been building The Natural Products Database from The University of Panama, Republic of Panama: CIFPMA. This dataset was first disclosed in 2017 [16].

#### Contents

The first disclosure of CIFPMA contained 354 compounds [16] and recently was updated to 454 molecules [17]. CIFPMA has compounds that have been tested biologically under more than 25 *in vitro* and *in vivo* bioassays. Examples of target therapeutic indications are anti-HIV, antioxidants and anticancer.

#### Accessibility & searching capabilities

A website is under construction. Currently, the chemical structures would be available upon request.

#### Diversity analysis & other applications

The content, diversity analysis, as systematic structure–structure activity relationship studies of compounds in CIFPMA have been reported [16,17]. The first version with 354 molecules was compared with NuBBE_DB_, molecules from the Traditional Chinese Medicine database, compounds with drug indications in ChEMBL and other reference libraries of NPs. It was concluded that metabolites in CIFPMA have large scaffold diversity and also has several unique scaffolds. The high scaffold diversity is in agreement with the broad range of biological activities [16]. The most recent version of CIFPMA was compared with other NPs databases including NuBBE_DB_ and BIOFACQUIM, drugs approved for clinical use, and synthetic compounds [17]. The comparison was made based on drug-like physicochemical properties, structural fingerprints and molecular scaffolds. It was concluded that NP databases have higher structural complexity than synthetic compounds. It was also concluded that compounds from synthetic origin have a larger proportion of aromatic atoms [17].

### UNIIQUIM

#### Developers

For more than 5 years, the Informatics Unit of the Institute of Chemistry (UNIIQUIM, for its acronym in Spanish) of the National Autonomous University of Mexico (UNAM) has been assembling and curating an open database with NP from Mexico, mainly isolated at published by researchers of the Natural Products Department of the Institute of Chemistry, UNAM.

#### Contents

This is a database intended to collect part of the large biodiversity of Mexico that has been published by the Natural Products Department of the Institute of Chemistry, UNAM. Compounds in UNIIQUIM are NP isolated in Mexico from plants, fungi, marine organisms, and insects found in Mexico. The total number of compounds is not totally clear from the website that is available only in Spanish.

Compounds in UNIIQUIM are annotated with chemical and biological data, when available. Chemical information includes molecular formula, IUPAC names, chemical abstract service (CAS) number and the chemical structure. Each compound record is linked to the reported biological activity, if reported in the publication source.

#### Accessibility & searching capabilities

UNIIQUIM database is accessible at the website interface that is currently available in Spanish (an English version will be released). It is not possible to download the entire database. The user can browse the contents by displaying either of two look-up tables: list of chemical compounds and list of organisms. The user can select the desired chemical compound or organism for specific information. It is also possible to search the database by bibliographic information.

#### Diversity analysis & other applications

To the best of our knowledge there are no reports of published applications of UNIIQUIM. It is anticipated that the database will be cited in the near future.

### BIOFACQUIM

#### Developers

For the past 2 years, the Computer-Aided Design at the School of Chemistry group (DIFACQUIM, for its acronym in Spanish) at UNAM is building and curating a NP database containing compounds isolated in Mexico. The final goal is capturing, as much as possible, the Mexican biodiversity.

#### Contents

The first version of BIOFACQUIM was released in 2019 and contained 423 molecules gathered from publications of the School of Chemistry for a 10-year period [18]. The same year, the database was updated with 148 structures to reach 553 compounds including molecules isolated not only in that institution but also by research groups in other Mexican institutions. As other NP databases discussed herein, BIOFACQUIM continue to be updated. Most of the compounds in BIOFACQUIM were isolated from plant, bacteria, and Mexican propolis.

Molecules in BIOFACQUIM are annotated with the chemical name and structure, bibliographic information, kingdom, genus, and species of the NP and geographical location of the collection. If the biological information is included in the original publication, the activity data is included in the compound record.

#### Accessibility & searching capabilities

The first version of BIOFACQUIM is accessible and searchable at the “BIOFACQUIM Explorer” website. It is also available at ZINC 15 and is part of the COCONUT database [10]. The second version of BIOFACQUIM is freely accessible at Figshare [19].

#### Diversity analysis & other applications

A comprehensive diversity analysis of the first release of BIOFACQUIM was published recently, along with the disclosure of the database itself [18]. It was concluded that compounds in this database have a broad coverage of the chemical space, overlapping with drug-like space as compared with approved drugs. Furthermore, the analysis also revealed structures with high chemical similarity to drug in clinical use. Recently, the chemical fragments in BIOFACQUIM were compared with those fragments available in ChEMBL 25, and a therein constructed assembled dataset with 169,839 unique structures of NPs [19]. It was concluded that, as expected, the chemical diversity of BIOFACQUIM increased in terms of chemical scaffolds and structural fingerprints relative to the first version. It was also concluded that, despite the relative few number of compounds in BIOFACQUIM as compared with the reference databases, there are a significant number of compounds, scaffolds and functional groups in BIOFACQUIM that are not present in the reference datasets [19].

## Toward LANaPD

Herein it is proposed building a unified database of NPs that represent the biodiversity of Latin America. Challenging tasks that can be overcome, one more difficult than others are discussed hereunder. Recent guidelines to assemble databases of NP have been published, in particular when intended to be used in virtual screening [9].

### Collection & standardization

The first step toward creating LANaPD is putting together all NP databases, processing and curating them using standard protocols. Although this step is not straightforward, it is feasible. It would be advisable that a research group would be in charge of this endeavor using publicly accessible tools and scripts or workflows available in public repositories such as Github. Examples of freely accessible workflows to curate compound database are available COCONUT database (*vide supra*) is an example of a large-scale database assembled and curated from several different sources around the world [10]. However, as discussed above, COCONUT is not focused on specific geographical regions and it does not contain all public databases from Latin America.

### Accessibility

Ideally, LANaPD can be made accessible to the public. This can be done by generating a web server dedicated to the database following the Findable, Accessible, Interoperable and Reusable (FAIR) principles [20]. Another option to deploy the database is using a public repository such as Figshare (https://figshare.com/) or ZENDO (https://zenodo.org/) where uploads are assigned a digital object identifier making them easily and uniquely citeable. LANaPD could be also accessible through other major databases broadly used so far like the ZINC 15 database. NP databases such as NuBBE_DB_, BIOFACQUIM, and AfroDB, for example, are accessible through ZINC 15 database.

### Maintenance

Updating and maintaining compound databases is of critical importance for the sustained and timely use of the information. This is also a challenging step, in particular for public databases, because of issues of sustained funding that experience basically all research groups and consortiums. For instance, it is well known that several web servers in the public domain are discontinued after certain time. In the NPs area an example is the Universal Natural Products Database [13] that, at that time, was the largest noncommercial and openly available database and contained 197,201 NPs from plants, animals, and microorganisms. The website hosting the database is no longer accessible. One of the workaround to address this problem is making use of repositories with permanent link with a digital object identifier number such as Fighshare or ZENDO (*vide supra*). Else, be successful in getting financial resources to sustain the website. An excellent example of such open compound database is ZINC 15 hosted by a research group at the University of California in San Francisco. Other examples of public databases with sustained financial support are PubChem, ChEMBL, and DrugBank.

Table 1 summarizes the NP databases currently developed and published in Latin American countries and that can serve as a starting point toward LANaPD. As stated in this report, research groups from these and other countries are invited to participate in this joint effort.

**Table 1.** Overview of current natural product databases developed in Latin America.

Database	Country	Brief description	URL
NuBBE_DB_	Brazil	Over 2000 secondary metabolites of plants, fungi, insects, marine organisms and bacteria.	https://nubbe.iq.unesp.br/portal/nubbe-search.html (English and Portuguese)
CIFPMA	Panama	Over 450 natural products from Panama	Online database under construction
UNIQUIM	Mexico	Compounds from plants, fungi, marine organisms and insects	https://uniiquim.iquimica.unam.mx/ (Spanish only)
BIOFACQUIM	Mexico	Over 550 compounds from Mexican biodiversity mainly from plants, fungi and Mexican propolis	First version at BIOFACQUIM Explorer: https://biofacquim.herokuapp.com/ (English only) Second version: http://doi.org/10.6084/m9.figshare.11312702

## Conclusion

In-line with the continued significance of NP to drug discovery and the accessibility of informatics resources, Latin American countries are developing compound databases with compounds available in their geographical region. Such efforts are part of a larger and global scale of research groups developing NP databases available in the public domain, representing the biodiversity of other countries. Thus far, Brazil, Mexico, and Panama have developed their databases releasing to the public the compounds and/or information of their contents. Other countries such as Colombia, Perú, and El Salvador are also currently building large databases that will be released soon. The largest database this far is NuBBE_DB_ from Brazil with over 2000 compounds. Building and maintaining all these databases are ongoing projects and the databases continue to grow as collecting the large biodiversity available in Latin American countries is challenging. It is expected that, putting all resources together in a single and unified compound database that can be called LANaPD, will have a significant contribution to NP research and NP-based drug discovery not only in Latin America but worldwide.

## Future perspective

Over the next 5 years, it is anticipated that the first version of the Latin American Natural Products Database: LaNaPD, proposed in this report, will be developed and be up and running. During this time frame, it is also expected that the contents of chemical structures and fragments, diversity and coverage of chemical space will be characterized using well established and innovative chemoinformatic techniques [21,22]. Over the following 5 years, it is envisioned that more countries in Latin America integrate their databases in LANaPD. It is also expected that such a unified database is being actively used in data mining, virtual screening, and other artificial intelligence applications [23].

**Summary points**

- Natural products (NPs) and compound databases have a significant impact on drug discovery.
- Countries around the world are building NP databases using chemoinformatics resources.
- The main goal of this report is to discuss the recent progress of countries in Latin America to generate and analyze compound databases of NPs.
- Currently, Brazil, Mexico, and Panama have developed databases releasing to the public the compounds and/or information of their contents.
- Colombia, Perú, and El Salvador, among other countries, are building NP databases that will be released soon.
- It is proposed to join efforts and assemble a unified Latin American Natural Products Database.
- It is anticipated that Latin American Natural Products Database will have a significant contribution to NP research not only in Latin America but worldwide.
